# Temporal trends in vaccination coverage in the first year of life in Brazil

**DOI:** 10.1590/1984-0462/2024/42/2023020

**Published:** 2023-10-23

**Authors:** Ana Beatriz Batista Neves, Lucas Emanuel de Oliveira Silva, Gabriela Morais Celestino Amaral, Maria Rosa da Silva, Claudio José dos Santos

**Affiliations:** a Universidade Estadual de Ciências da Saúde de Alagoas, Maceió, AL, Brazil.; b Universidade de São Paulo, São Paulo, SP, Brazil.

**Keywords:** Time series studies, Epidemiological monitoring, Immunization programs, Vaccination coverage, Immunization, Estudos de séries temporais, Monitoramento epidemiológico, Programas de imunização, Cobertura vacinal, Imunização

## Abstract

**Objective:**

The aim of this study was to analyze the temporal trends in vaccination coverage (VC) during the first year of life of children in Brazil.

**Methods:**

Data on VC for the first year of life from 2011 to 2020 for Bacille Calmette-Guerin (BCG), hepatitis B, polio, pentavalent, and triple viral vaccines at the national, regional, and state levels were obtained from the Information System of the National Immunization Program. Trends were analyzed using Prais-Winsten generalized linear regression models and average annual percent change (APC) estimates.

**Results:**

Decreasing trends were observed for the BCG (APC −3.58%; p<0.05), pentavalent (APC −4.10%; p<0.05), polio (APC −2.76%; p<0.05), and triple viral (APC −2.56%; p<0.05) vaccines in the country. Hepatitis B vaccine was the only vaccine that displayed stationary behavior (APC −4.22%; p>0.05). During the study period, no increasing trends were observed in any territory or vaccine.

**Conclusions:**

This study shows a recent significant reduction and decreasing trends in VC during the first year of life of children in Brazil, indicating the need for interventions to curb this ongoing phenomenon and to recover acceptable VC rates in the country.

## RESUMO

**Objetivo:**

Analisar a tendência temporal da cobertura vacinal (CV) em crianças com idade menor ou igual a um ano no Brasil.

**Métodos:**

Foram empregados dados da CV no primeiro ano de vida de 2011 a 2020 referentes às vacinas BCG, hepatite B, poliomielite, pentavalente e tríplice viral, obtidos do Sistema de Informação do Programa Nacional de Imunizações (SI-PNI). Os dados estão agregados em nível nacional, regional e estadual. Para a análise de tendência foi utilizado o modelo de regressão linear generalizado de Prais-Winsten e foi calculada a variação percentual média anual (APC).

**Resultados:**

As tendências de CV para as vacinas BCG (APC −3,58%; p<0,05), pentavalente (APC −4,10%; p<0,05), poliomielite (APC −2,76%; p<0,05) e tríplice viral (APC −2,56%; p<0,05) foram decrescentes no país. Apenas para a CV da vacina contra hepatite B foi identificado comportamento estacionário (APC −4,22%; p>0,05). Nenhum território ou vacina apresentou tendência crescente para cobertura vacinal no período estudado no Brasil.

**Conclusões:**

Este estudo alerta sobre a redução expressiva das CV no primeiro ano de vida nos últimos anos no Brasil e sua tendência decrescente, sendo imperativa a adoção de intervenções com o fim de frear o fenômeno em curso e de resgatar níveis aceitáveis de CV no país.

## INTRODUCTION

Brazil is well known worldwide for its successful vaccination programs that offer free universal immunization to the entire population.^1^ The National Immunization Program (NIP) was established in 1973 to coordinate all actions related to immunization, primarily aiming to eliminate smallpox, polio, measles, and tetanus.^2^ The NIP is now recognized as one of the most effective health initiatives globally for preventing and reducing mortality from communicable diseases.^3^

The direct advantages of vaccines are extensively recognized. Immunization protection can help prevent medical expenditures. Additionally, vaccination has a positive correlation with cognition and school performance, indicating benefits in terms of long-term economic productivity.^4^ Finally, there is the occurrence of heterologous immunity, where immunization or previous exposure to one pathogen provides some level of protection against a different, unrelated pathogen.^4^

Despite the benefits of vaccination and the historical institutional efforts to control and eliminate infectious diseases, Brazil has experienced declining vaccination coverage (VC) rates.^5^ This is particularly concerning for children who are more vulnerable to infectious diseases. The absence of vaccination increases the risk of re-emergence of previously controlled or eradicated diseases such as the resurgence of measles^6^ and the possible resurgence of polio.^7^ Although immunogens for both diseases are part of the national vaccination schedule in Brazil, there have been successive decreases in VC since 2015, as observed in the case of other immunizing agents.^8^

The NIP in Brazil mandates a vaccination schedule for children under 1 year of age, which includes a range of vaccines such as hepatitis B, rotavirus, bacterial triple vaccine, *Haemophilus influenzae* B, polio, pneumococcal conjugate, meningitis, yellow fever, hepatitis A, and triple viral vaccine.^9^ Receiving these vaccines in the first year of life provides children with immunological protection against these diseases, which can help prevent severe complications, hospitalizations, and even death.^10^ In addition, vaccination in this age group contributes to reducing the number of cases of infectious diseases in the entire population and also protecting those who cannot receive vaccines for health reasons.^11^

Due to the variety of vaccines offered and updates to the program over time, studies analyzing VC for this age group often consider different types of vaccines.^12^ Therefore, this study aims to examine the trend in Bacille Calmette-Guerin (BCG), hepatitis B, polio, pentavalent, and triple viral VC among Brazilian children in the first year of life, as well as in different geographic regions and states, from 2011 to 2020.

## METHOD

This is an ecological time series study that utilized secondary data recorded in the Information System of the National Immunization Program (IS-NIP). The units of analysis were 27 Brazilian states and 5 geographic regions.

This study utilized data on VC in the first year of life for vaccines aimed at preventing severe forms of tuberculosis such as meningeal and miliary tuberculosis, where a single dose of BCG is recommended after birth. The study also used data on hepatitis B vaccination, which is administered in the first 30 days of life, the third dose of injectable polio vaccine administered at 6 months of age, the third dose of pentavalent vaccine (which includes diphtheria, tetanus, pertussis, *Haemophilus influenzae* B, and hepatitis B) recommended at 6 months of age, and the first dose of the triple viral vaccine (which includes mumps, measles, and rubella) administered at 12 months of age.

The VC data for each territory and each vaccine were obtained from the IS-NIP via the website of the Department of Informatics of the Unified Health System of the Ministry of Health (DATASUS).^13^ The numerator of this indicator represents the number of doses administered to the target population in a certain period and place, while the denominator represents the total target population for the vaccine. The quotient is then multiplied by 100.^14^ Equation 1 illustrates the calculation of this indicator:


(1) $$VC=\frac{Number\;of\;doses\;given\;to\;the\;target\;population}{Total\;target\;population}x100$$

The Ministry of Health advocates the following VC percentage targets in Brazilian territory:

- BCG: 90%
- Hepatitis B: 95% for the first dose and 90% for the third dose
- Polio: 95%
- Pentavalent: 95%
- Triple viral: 95%

The study examined data from a specific time period for several vaccines. Specifically, the data from the last 10 years were analyzed for the BCG, polio, and triple viral vaccines, from 2011 to 2020. The last 7 years were analyzed for the hepatitis B vaccine, from 2014 to 2020, and the last 8 years were analyzed for the pentavalent vaccine, from 2013 to 2020. Data from 2021 were not included in the analysis because, at the time of data collection, the reporting of doses administered during that year in the IS-NIP system by the municipalities was still ongoing.

A generalized linear regression model that employed the Prais-Winsten method was used to examine changes in VC indicators over time. A level of statistical significance of p<0.05 was used to determine the significance of the results. An increasing trend was identified by a positive and statistically significant model coefficient (β>0, p<0.05), while a decreasing trend was identified by a negative and statistically significant model coefficient (β<0, p<0.05). A stationary trend was identified when there was no statistical significance (p>0.05). In addition, the average annual percent change (APC) and corresponding 95% confidence intervals (CIs) were calculated.

The data analyses were conducted using the Stata software version 16. Consistent with scientific transparency guidelines,^15^ all materials necessary for replicating the analyses, such as computational data and scripts, have been made publicly available on the Open Science Framework repository.

## RESULTS

Table 1 presents a comprehensive summary of the Prais-Winsten regression model estimates for the BCG, hepatitis B, polio, pentavalent, and triple viral vaccines administered in Brazil and geographic regions, for the period spanning from 2011 to 2020. Furthermore, Tables 2 to 6 provide a detailed breakdown of these estimates, specifically focusing on the states within the North, Northeast, Southeast, South, and Midwest regions, respectively.

**Table 1:** Prais-Winsten regression estimates for vaccination coverage rates in the first year of life of Bacille Calmette-Guerin (2011–2020), hepatitis B (2014–2020), pentavalent (2013–2020), polio (2011–2020), and triple viral (2011–2020) vaccines in Brazil and geographic regions.

Unit	BCG		Hepatitis B		Pentavalent		Polio		Triple viral	
	β	APC (95%CI)	β	APC (95%CI)	β	APC (95%CI)	β	APC (95%CI)	β	APC (95%CI)
Brazil	−0.02*	−3.58 (−5.89; −1.20)	−0.02	−4.22 (−8.31; 0.06)	−0.02*	−4.10 (−5.42; −2.76)	−0.01*	−2.76 (−3.80; −1.71)	−0.01*	−2.56 (−4.21; −0.87)
North	−0.02*	−3.92 (−5.20; −2.64)	−0.00	−1.94 (−5.99; 2.29)	−0.02*	−4.58 (−5.38; −3.77)	−0.02*	−4.03 (−5.48; −2.55)	−0.02*	−3.90 (−6.28; −1.46)
Northeast	−0.02*	−3.83 (−6.33; −1.27)	−0.01	−2.92 (−7.17; 1.52)	−0.02*	−4.27 (−6.38; −2.11)	−0.01*	−3.11 (−4.57; −1.63)	−0.01*	−2.76 (−4.66; −0.81)
Southeast	−0.02*	−3.85 (−7.19; −0.40)	−0.03	−6.67 (−13.34; 0.51)	−0.02*	−4.02 (−5.76; −2.25)	−0.01*	−2.58 (−3.54; −1.61)	−0.01*	−2.14 (−3.14; −0.84)
South	−0.01*	−2.44 (−3.75; −1.12)	−0.02*	−3.42 (−4.68; −2.14)	−0.02*	−3.65 (−4.43; −2.86)	−0.01*	−1.60 (−2.31; −0.88)	−0.01	−1.78 (−3.68; 0.15)
Midwest	−0.01*	−3.24 (−5.24; −1.20)	−0.01	−3.23 (−6.80; 0.46)	−0.02*	−5.17 (−6.18; −4.15)	−0.01*	−2.80 (−4.25; −1.32)	−0.01*	−3.19 (−5.34; −0.98)

* p-value<0.05; BCG: Bacille Calmette-Guerin; β: regression coefficient; APC: annual percent change (%); 95%CI: 95% confidence interval.

Source: Authors through data obtained from NIP/SUS; DATASUS.

**Table 2:** Prais-Winsten regression estimates for vaccination coverage rates in the first year of life of Bacille Calmette-Guerin (2011–2020), hepatitis B (2014–2020), pentavalent (2013–2020), polio (2011–2020), and triple viral (2011–2020) vaccines in Federative Units in North region.

FU	BCG		Hepatitis B		Pentavalent		Polio		Triple viral	
	β	APC (95%CI)	β	APC (95%CI)	β	APC (95%CI)	β	APC (95%CI)	β	APC (95%CI)
AC	−0.01*	−3.32 (−5.67; −0.92)	0.04	10.58 (−6.83; 31.24)	0.00	−0.74 (−2.29; 0.83)	−0.02*	−4.38 (−6.87; −1.83)	−0.02*	−3.83 (−5.97; −1.64)
AP	−0.01	−2.32 (−4.68; 0.10)	−0.01	−1.39 (−3.80; 1.08)	−0.05*	−10.30 (−15.18; −5.14)	−0.03*	−6.20 (−9.92; −2.32)	−0.02*	−4.36 (−7.27; −1.36)
AM	−0.02*	−3.69 (−5.07; −2.28)	−0.01	−2.49 (−5.08; 0.18)	−0.02*	−3.62 (−5.88; −1.30)	−0.01*	−3.02 (−5.78; −0.18)	−0.01*	−2.64 (−5.04; −0.19)
PA	−0.02*	−5.54 (−7.53; −3.50)	−0.02	−3.64 (−11.13; 4.49)	−0.03*	−6.27 (−8.62; −3.86)	−0.03*	−5.64 (−8.50; −2.69)	−0.02*	−5.59 (−9.12; −1.93)
RO	−0.17*	−3.84 (−7.03; −0.53)	−0.02*	−5.58 (−7.35; −3.78)	−0.01	−1.88 (−5.24; 1.59)	−0.01	−2.21 (−4.63; 0.28)	−0.01	−1.83 (−4.92; 1.36)
RR	0.00	0.11 (−2.97; 3.29)	0.03	6.51 (−4.54; 18.85)	−0.01	−2.60 (−7.36; 2.40)	−0.01	−2.32 (−4.97; 0.39)	−0.01	−2.75 (−6.45; 1.10)
TO	0.00	0.70 (−0.39; 1.81)	0.01	3.30 (−0.82; 7.60)	−0.01*	−2.97 (−3.75; −2.18)	−0.01*	−1.40 (−2.10; −0.69)	−0.01	−1.38 (−3.34; 0.61)

* p-value<0.05; FU: Federative Units; AC: Acre; AP: Amapá; AM: Amazonas; PA: Pará; RO: Rondônia; RR: Roraima; TO: Tocantins; BCG: Bacille Calmette-Guerin; β: regression coefficient; APC: annual percent change (%); 95%CI: 95% confidence interval.

Source: Authors through data obtained from NIP/SUS; DATASUS.

**Table 3:** Prais-Winsten regression estimates for vaccination coverage rates in the first year of life of Bacille Calmette-Guerin (2011–2020), hepatitis B (2014–2020), pentavalent (2013–2020), polio (2011–2020), and triple viral (2011–2020) vaccines in Federative Units in Northeast region.

FU	BCG		Hepatitis B		Pentavalent		Polio		Triple viral	
	β	APC (95%CI)	β	APC (95%CI)	β	APC (95%CI)	β	APC (95%CI)	β	APC (95%CI)
AL	−0.02	−3.56 (−8.68; 1.84)	−0.02	−4.30 (−11.50; 3.48)	−0.01	−2.01 (−4.25; 0.28)	−0.01	−1.64 (−3.57; 0.34)	0.00	−0.41 (−3.30; 2.56)
BA	−0.02*	−3.71 (−4.92; −2.48)	−0.01*	−2.29 (−3.86; −0.69)	−0.02*	−4.42 (−6.31; −2.48)	−0.02*	−3.84 (−5.38; −2.26)	−0.01*	−3.29 (−5.79; −0.73)
CE	−0.02	−4.72 (−10.10; 0.97)	−0.02	−5.39 (−13.92; 4.00)	−0.01	−2.72 (−6.59; 1.30)	0.00	−1.00 (−3.30; 1.35)	−0.01	−1.55 (−4.57; 1.56)
MA	−0.02*	−5.50 (−8.83; −2.04)	−0.02	−5.12 (−12.21; 2.55)	−0.04*	−7.79 (−10.87; −4.61)	−0.02*	−5.24 (−7.30; −3.14)	−0.02*	−5.13 (−7.98; −2.18)
PB	−0.02*	−4.73 (−7.89; −0.70)	−0.02	−3.70 (−11.11; 4.32)	−0.01*	−3.14 (−4.73; −1.52)	−0.01*	−2.59 (−4.40; −0.75)	−0.01	−1.73 (−4.15; 0.76)
PE	−0.01*	−3.01 (−5.22; −0.75)	0.00	0.17 (−4.47; 5.05)	−0.02*	−4.73 (−7.63; −1.73)	−0.02*	−3.50 (−5.27; −1.70)	−0.01*	−2.32 (−3.68; −0.94)
PI	−0.01*	−2.26 (−4.16; −0.32)	−0.01	−1.23 (−6.60; 4.45)	−0.02*	−4.13 (−6.91; −1.28)	−0.01*	−2.94 (−5.18; −0.66)	−0.01*	−2.45 (−4.59; −0.26)
RN	−0.02*	−4.23 (−6.84; −1.55)	0.00	−1.02 (−8.89; 7.53)	−0.02*	−3.85 (−6.45; −1.18)	−0.01*	−3.28 (−5.97; −0.51)	−0.01*	−2.95 (−5.30; −0.54)
SE	−0.01	−2.18 (−5.54; 1.31)	−0.03*	−6.77 (−10.99; −2.35)	−0.02*	−3.70 (−5.56; −1.81)	−0.02*	−3.49 (−4.96; −1.98)	−0.01*	−2.33 (−3.81; −0.84)

* p-value<0.05; FU: Federative Units; AL: Alagoas; BA: Bahia; CE: Ceará; MA: Maranhão; PB: Paraíba; PE: Pernambuco; PI: Piauí; RN: Rio Grande do Norte; SE: Sergipe; BCG: Bacille Calmette-Guerin; β: regression coefficient; APC: annual percent change (%); 95%CI: 95% confidence interval.

Source: Authors through data obtained from NIP/SUS; DATASUS.

**Table 4:** Prais-Winsten regression estimates for vaccination coverage rates in the first year of life of Bacille Calmette-Guerin (2011–2020), hepatitis B (2014–2020), pentavalent (2013–2020), polio (2011–2020), and triple viral (2011–2020) vaccines in Federative Units in Southeast region.

FU	BCG		Hepatitis B		Pentavalent		Polio		Triple viral	
	β	APC (95%CI)	β	APC (95%CI)	β	APC (95%CI)	β	APC (95%CI)	β	APC (95%CI)
ES	−0.01*	−2.52 (−4.06; −0.95)	−0.01	−2.80 (−6.24; 0.76)	−0.02*	−4.87 (−6.30; −3.42)	−0.01*	−3.02 (−3.96; −2.06)	−0.01*	−2.23 (−3.40; −1.04)
MG	−0.01*	−2.15 (−3.36; −0.92)	−0.01	−1.70 (−5.06; 1.78)	−0.02*	−3.48 (−4.54; −2.41)	−0.01*	−1.83 (−2.74; −0.91)	−0.01*	−1.46 (−2.74; −0.16)
RJ	−0.03	−6.19 (−12.16; 0.18)	−0.05	−11.06 (−21.89; 1.26)	−0.03*	−7.37 (−14.15; −0.04)	−0.03*	−6.50 (−10.54; −2.28)	−0.02	−3.77 (−7.53; 0.15)
SP	−0.02*	−4.00 (−7.57; −0.30)	−0.03	−7.58 (−15.38; 0.93)	−0.01*	−3.31 (−4.45; −2.15)	−0.01*	−1.96 (−2.88; −1.04)	−0.01*	−1.93 (−3.13; −0.70)

* p-value<0.05; FU: Federative Units; ES: Espírito Santo; MG: Minas Gerais; RJ: Rio de Janeiro; SP: São Paulo; BCG: Bacille Calmette-Guerin; β: regression coefficient; APC: annual percent change (%); 95%CI: 95% confidence interval.

Source: Authors through data obtained from NIP/SUS; DATASUS.

**Table 5:** Prais-Winsten regression estimates for vaccination coverage rates in the first year of life of Bacille Calmette-Guerin (2011–2020), hepatitis B (2014–2020), pentavalent (2013–2020), polio (2011–2020), and triple viral (2011–2020) vaccines in Federative Units in South region.

FU	BCG		Hepatitis B		Pentavalent		Polio		Triple viral	
	β	APC (95%CI)	β	APC (95%CI)	β	APC (95%CI)	β	APC (95%CI)	β	APC (95%CI)
PR	−0.01*	−2.16 (−3.21; −1.09)	−0.02*	−4.96 (−6.60; −3.31)	−0.01*	−3.33 (−3.99; −2.67)	−0.01*	−1.91 (−2.72; −1.09)	−0.01	−2.05 (−4.19; 0.13)
RS	−0.01*	−2.33 (−3.79; −0.85)	−0.01	−1.28 (−2.97; 0.45)	−0.02*	−3.97 (−4.27; −3.67)	−0.01*	−1.51 (−2.58; −0.43)	−0.01	−1.51 (−3.47; 0.49)
SC	−0.01*	−3.17 (−5.09; −1.22)	−0.04*	−9.54 (−13.47; −5.44)	−0.02*	−3.68 (−5.72; −1.60)	0.00*	−1.14 (−1.58; −0.70)	−0.01	−1.71 (−3.40; 0.02)

* p-value<0.05; FU: Federative Units; PR: Paraná; RS: Rio Grande do Sul; SC: Santa Catarina; BCG: Bacille Calmette-Guerin; β: regression coefficient; APC: annual percent change (%); 95%CI: 95% confidence interval.

Source: Authors through data obtained from NIP/SUS; DATASUS.

**Table 6:** Prais-Winsten regression estimates for vaccination coverage rates in the first year of life of Bacille Calmette-Guerin (2011–2020), hepatitis B (2014–2020), pentavalent (2013–2020), polio (2011–2020), and triple viral (2011–2020) vaccines in Federative Units in Midwest region.

FU	BCG		Hepatitis B		Pentavalent		Polio		Triple viral	
	β	APC (95%CI)	β	APC (95%CI)	β	APC (95%CI)	β	APC (95%CI)	β	APC (95%CI)
DF	−0.01	−2.46 (−5.47; 0.64)	−0.01*	−3.33 (−6.49; −0.06)	−0.01	−2.67 (−8.30; 3.30)	−0.01	−1.55 (−4.64; 1.64)	−0.01	−1.78 (−4.21; 0.72)
GO	−0.02*	−4.59 (−5.95; −3.21)	−0.01	−1.40 (−4.69; 2.01)	−0.03*	−5.84 (−6.62; −5.05)	−0.02*	−3.69 (−4.91; −2.46)	−0.02*	−4.72 (−7.22; −2.15)
MS	−0.01	−3.20 (−9.65; 3.70)	−0.02	−5.04 (−14.29; 5.12)	−0.03*	−5.74 (−7.94; 3.49)	−0.01	−2.20 (−6.35; 2.13)	−0.01	−1.98 (−6.23; 2.46)
MT	−0.01*	−2.33 (−4.04; −0.59)	−0.02*	−4.26 (−7.39; 1.03)	−0.02*	−4.59 (−6.54; −2.61)	−0.01*	−2.70 (−3.96; −1.42)	−0.01	−2.48 (−4.90; 0.01)

* p-value<0.05; FU: Federative Units; DF: Distrito Federal, GO: Goiás; MS: Mato Grosso do Sul; MT: Mato Grosso; BCG: Bacille Calmette-Guerin; β: regression coefficient; APC: annual percent change (%); 95%CI: 95% confidence interval.

Source: Authors through data obtained from NIP/SUS; DATASUS.

In the case of BCG, a decreasing trend in VC was observed at both the national level (APC −3.58%; 95%CI −5.89; −1.20) and the regional level. The steepest decline was observed in the North region (APC −3.92%; 95%CI −5.20; −2.64), followed by the Southeast (APC −3.85%; 95%CI −7.19; −0.40), Northeast (APC −3.83%; 95%CI −6.33; −1.27), Midwest (APC −3.21%; 95%CI −5.24; −1.20), and South (APC −2.44%; 95%CI −3.75; −1.12). At the state level, the steepest decline in VC was observed in Pará (APC −5.54%; 95%CI −7.53; −3.50).

Hepatitis B vaccine coverage presented a stationary trend at both the national level (APC −4.22%; 95%CI −8.31; 0.06) and the regional level, except for the South region that showed a decreasing trend (APC −3.42%; 95%CI −4.68; −2.14). The largest decline was recorded in Santa Catarina (APC −9.54%; 95%CI −13.47; 5.44).

The figures for the pentavalent vaccine indicated a similar pattern to that observed for BCG. Between 2013 and 2020, there was a reduction in VC in both the country as a whole (APC −4.10%; 95%CI −5.42; −2.76) as well as all geographic regions. In this case, the largest decline occurred in the Midwest (APC −5.17%; 95%CI −6.18; −4.15) followed by the North (APC −4.58%; 95%CI −5.38; −3.77), Northeast (APC −4.27%; 95%CI −6.38; −2.11), Southeast (APC −4.02%; 95%CI −5.76; −2.25), and South (APC −3.65%; 95%CI −4.43; −2.86). The state of Amapá presented the greatest decrease in VC (APC −10.30%; 95%CI −15.18; −5.14).

Polio vaccine coverage also showed a downward trend in both the country as a whole (APC −2.76%; 95%CI −3.80; −1.71) as well as the regions. The steepest decline was observed in the North (APC −4.03%; 95%CI −5.48; −2.55), followed by Northeast (APC −3.11%; 95%CI −4.57; −1.63), Midwest (APC −2.80%; 95%CI −4.25; −1.32), Southeast (APC −2.58%; 95%CI −3.54; −1.61), and South (APC −1.60%; 95%CI −2.31; −0.88). At the state level, Amapá had the steepest decline in VC at the state level (APC −6.20; 95%CI −9.92; −2.32).

Finally, the triple viral vaccine also showed a decreasing trend in coverage at the national level (APC −2.56%; 95%CI −4.21; −0.87). Among the regions, the North stood out with the largest decline (APC −3.90%; 95%CI −6.28; −1.46), followed by the Midwest (APC −3.19%; 95%CI −5.34; −0.98), Northeast (APC −2.76%; 95%CI −4.66; −0.81), and Southeast (APC −2.14%; 95%CI −3.14; −0.84). The South was the only region that showed a stationary trend during the analyzed period (APC −1.78%; 95%CI −3.68; 0.15). At the state level, the largest decline was again recorded in Amapá (APC −4.36; 95%CI −7.27; −1.36).

**Figure 1:**
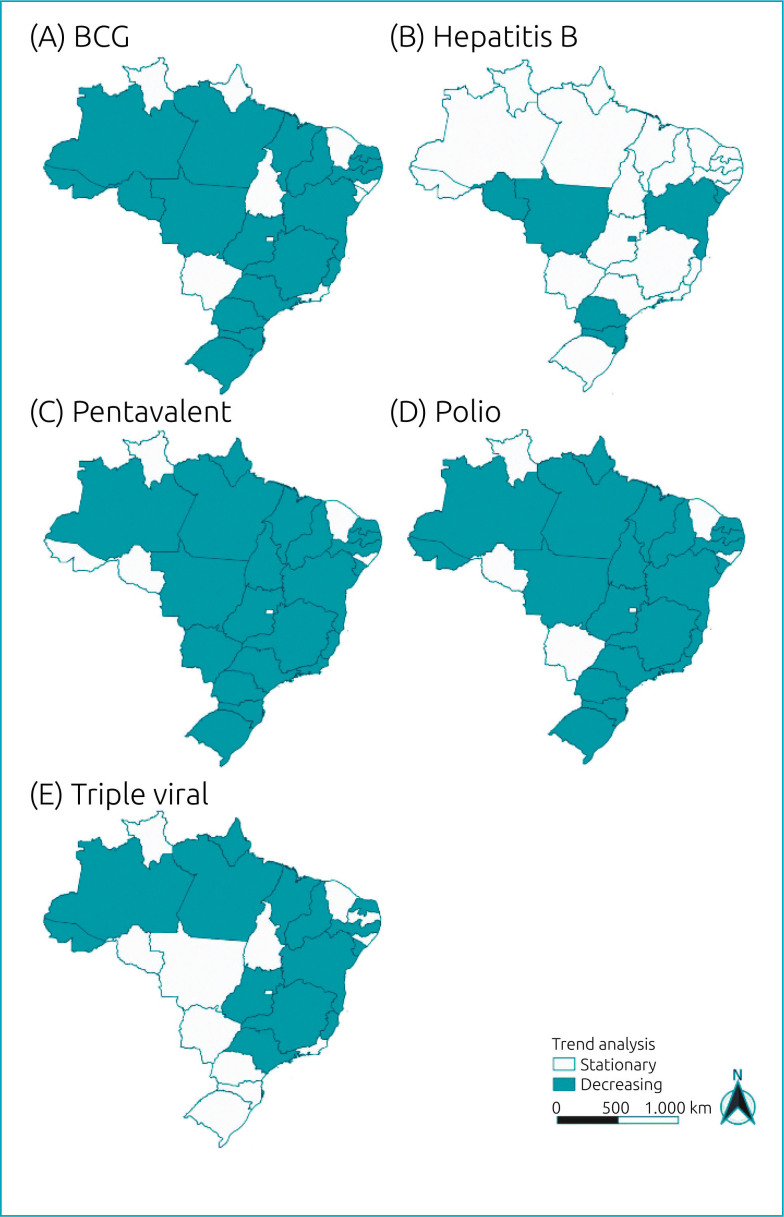
Trend analysis of vaccination coverage, by state, for the following vaccines in Brazil: (A) Bacille Calmette-Guerin (2011–2020), (B) hepatitis B (2014–2020), (C) pentavalent (2013–2020), (D) polio (2011–2020), and (E) triple viral (2011–2020).

Source: Authors through data obtained from NIP/SUS; DATASUS.

Upon analyzing the vaccination trend pattern at the state level, as depicted in Figure 1, it is evident that the most critical vaccination scenarios are the ones related to the pentavalent and polio vaccines. Both vaccines show a total of 21 states with a declining vaccination trend. Next, the BCG vaccine appears, which shows a reduction in 19 states. Conversely, the hepatitis B vaccine recorded the lowest number of territories (seven states) with a reduction in vaccination trend. It is noteworthy that none of the vaccines showed an increase, as the trends were either stationary or decreasing.

## DISCUSSION

The decline in VC is a recurring global phenomenon.^5^ In Brazil, a widespread decrease in vaccination rates began in 2015.^2,5^ This phenomenon has multiple causes, ranging from personal, political, and sociocultural aspects. Studies suggest that lower levels of education and lower social strata are associated with lower VC.^16^ Therefore, the lack of vaccination can contribute to perpetuating relationships of inequality.^17^

The declining trends in VC during the first year of life of children in Brazil, detected in this study, bear similarities to other findings in the literature.

In many Northeastern states, a reduction in the percentage of VC was detected. Queiroz et al.^18^ analyzed vaccination data for the basic immunization schedule for the first year of life in the capitals of the Brazilian Northeast and also detected a decrease in VC over 2013–2016.

Fonseca and Buenafuente^19^ identified low VC among children under 1 year of age in Roraima between 2013 and 2017. According to the authors, the decline was due to barriers present in the vaccination process.

A recent study by Souza et al.^20^ investigated the vaccination rates of children under 1 year old in Minas Gerais, Brazil from 2015 to 2020. The study analyzed trends in coverage and found that 8 out of the 28 health management units showed a decreasing trend in at least five of the seven immunobiological agents evaluated. The pentavalent vaccine, in particular, showed a decreasing trend in coverage in 60.71% of the localities analyzed.

Barcelos et al.^21^ also investigated VC in Brazil, focusing on children under 2 years old who were beneficiaries of the *Bolsa Família* Program. The study found low coverage in both the first and second year of life, with a social asymmetry in vaccination rates, as coverage was higher among children from families in the richest quintile and whose mothers had more than 9 years of schooling.

In contrast, Otero et al.^22^ analyzed vaccination rates in Curitiba, Paraná, from 2015 to 2020 and found significantly high coverage rates for at least six of the seven immunobiological agents recommended by the NIP to be given to children under 1 year old. The study found that the average coverage rates for all of the immunobiological agents were above 80% in the last 6 years.

Bahia, for example, was the only state analyzed which showed a reduction in coverage for all vaccines analyzed during the period. In this regard, Barata and Pereira^23^ address the relationship between social inequalities and vaccine coverage in children under 2 years of age in the city of Salvador, Bahia. The study was conducted through interviews with 699 mothers and analysis of their children's vaccination cards. The results showed that VC was lower among children whose mothers had lower levels of education and income, as well as those living in areas with worse socio-economic conditions.

In addition to social inequalities, the decline in VC can be attributed to others factors as the phenomenon of vaccine hesitancy that has gained attention in public discourse in recent years. Vaccine hesitancy refers to the delay in accepting or outright refusal of recommended vaccines, despite their availability in health services.^24^ It has become increasingly prevalent in Brazil, and a clear trend toward lower levels of confidence in vaccines has been observed in the country.^24^ Hesitant individuals comprise a diverse group, holding various degrees of indecision about specific vaccines or vaccination in general. While some may accept all vaccines but remain concerned about them, others may refuse or delay some vaccines but accept others, and still others may refuse all vaccines.^24^

The decrease in vaccine coverage can also be attributed to the resurgence of the anti-vaccine movement, which gained momentum in 1998 with the claim that vaccines were linked to the development of autism.^25^ With the rise of digital media, these groups now use social networks to disseminate conspiracy theories and fake news that are sensationalist and lack authorship, alleging that immunobiological agents are both ineffective and have the potential to cause death or serious health problems.^26^ This denialism discredits facts and overvalues conspiracy theories, which creates doubt about the safety of institutions in general. Consequently, trust in science, scientific societies, and public policies has declined, leading to greater hesitancy to participate in vaccination campaigns. To counteract the potential damage of conspiratorial discourses, health authorities have adopted different communication strategies with the public.^27^

The impact of the COVID-19 pandemic has also played a significant role in exacerbating existing health problems, including the decline in VC rates. Due to the burden on healthcare systems caused by the pandemic, health professionals were overwhelmed, and access to healthcare became more challenging. Additionally, with the public adopting social distancing recommendations and fearing social contact, healthcare service attendance was reduced.^28^ The present findings align with previous research that shows a decline in the coverage of 90% of the vaccines offered to children under the age of 1 in comparison to previous years, including the year 2020, which was heavily impacted by the pandemic.^29^

The statement suggests that the COVID-19 pandemic and the focus on emergency care and intensive care may have had a negative impact on vaccination efforts and contributed to the decline in VC in Brazil. Additionally, the inclusion of new vaccines, such as those for COVID-19, without effective campaigns to educate the public may have also contributed to vaccine resistance and refusal. This highlights the importance of implementing interventions to address these challenges and improve vaccination rates in the country.

Over time, institutional efforts have been made to address the issue of decreasing VC. One such effort was the transition of the IS-NIP from a method of tracking applied doses to a nominal record method.^14^ While this change improved the accuracy and quality of records, it also presented material and logistical challenges and required training of healthcare teams, which may have contributed to the lower registration of doses applied and affected the assessment of VC in certain areas.^30^

One limitation of this study pertains to the use of a secondary database, which may not be entirely reliable due to the involvement of multiple professionals and services in entering data on administered doses. However, it is worth noting that these data are also employed by official bodies to design and execute immunization policies in the country. Despite this limitation, the data remain useful in shedding light on VC trends in Brazil.

This study revealed a significant decrease in VC among children in their first year of life in Brazil, as well as a declining trend in VC for BCG, polio, pentavalent, and triple viral vaccines across most of the territories examined. Interventions are necessary to reverse this trend and increase acceptable VC rates in the country. Health agencies must engage in comprehensive social and political discussions on vaccination, as health education is essential to change attitudes toward vaccines. Additionally, combating misinformation is crucial to prevent it from becoming a threat to public health.

## Data Availability

The database that originated the article is available with the corresponding author.
